# Improving the Edible and Nutritional Quality of Roasted Duck Breasts through Variable Pressure Salting: Implications for Protein Anabolism and Digestion in Rats

**DOI:** 10.3390/foods13030402

**Published:** 2024-01-26

**Authors:** Ziwu Gao, Yinna Zhou, Dequan Zhang, Ruiyun Wu, Jiale Ma, Jinhua He, Zhenyu Wang

**Affiliations:** 1 Institute of Food Science and Technology, Chinese Academy of Agricultural Sciences, Beijing 100193, China; gzw96530@163.com (Z.G.); zyn12355@163.com (Y.Z.); wuruiyun814@163.com (R.W.); 17302258026@163.com (J.M.); 15102613432@163.com (J.H.); caasjgsmeat2021_6@126.com (Z.W.); 2 Integrated Laboratory of Processing Technology for Chinese Meat and Dish Products, Ministry of Agriculture and Rural Affairs, Beijing 100193, China

**Keywords:** variable pressure salting, in vivo digestion, water distribution, texture, protein structure

## Abstract

Variable pressure salting (VPS) is considered a novel salting approach to improve meat quality. This study aimed to investigate the effects of roasted duck’s edible and nutritional quality after VPS through serum biochemical indicators and in vivo digestion properties in rats. The results show that roasted duck after VPS led to an increase in the total protein content (57.24 g/L) and blood glucose levels (6.87 mmol/L), as well as a decrease in the blood urea nitrogen content (11.81 mmol/L), in rats. Compared to rats fed base diets and roasted duck after static wet salting (SWS), those ingesting roasted duck after VPS exhibited higher values of apparent protein digestibility (51.24%), pepsin activity (2.40 U/mg), and trypsin activity (389.80 U/mg). Furthermore, VPS treatment improved the textural properties and microstructure of duck breasts shown by a higher immobilized water relaxation area and more ordered protein structures (α-helixes and β-sheets). These improvements enhanced the protein anabolism capacity and in vivo digestion properties in rats. Therefore, VPS represents a beneficial salting method for promoting effective digestion and absorption in rats.

## 1. Introduction

Roasted duck is a well-known Chinese meat product celebrated for its distinctive flavor and rich nutritional profile [1]. Salting is a critical step in the processing of roasted duck, as it enhances the color, water retention, and flavor quality of the duck carcasses [2]. During the salting process, the brine enters the raw meat, increasing the osmotic pressure and promoting protein degradation and the release of taste substances (such as free amino acids, free fatty acids, and nucleotides) [3]. However, traditional static wet salting (SWS) has low salting efficiency and cannot respond to industrialized production. Additionally, vacuum tumbling and salting can destroy the shape of the raw meat, compromising the integrity of the whole carcass [4]. Therefore, it is necessary to develop an advanced salting method to improve the salting efficiency and keep the integrity of the raw meat. A novel approach, known as variable pressure salting (VPS), has recently been developed for salt duck carcasses [5]. VPS is applied to raw meat under alternating changes in the vacuum and pressure so that the raw meat and the brine make complete contact.

Previous studies showed that VPS is a potential method for industrial meat curing, as it improved the salting efficiency and raw meat quality [6]. For example, Jin et al. [7] found that VPS shortened the salting time, reduced the cooking loss, and improved the water retention of pork compared to SWS. Jiao et al. [5] reported that VPS reduced shear forces and improved the flavor profile of roasted duck breasts compared to static wet-salted duck breasts. Furthermore, during salting, Na^+^ and Cl^−^ disrupt the hydration layer on the protein’s surface, providing more binding sites for pepsin and improving digestibility [8]. Pulsed electric field processing might also improve the digestibility of meat proteins by inducing structural and microstructural changes in the protein and increasing access to meat protein hydrolysis sites [9]. It is reported that VPS treatment improved the in vitro digestibility of roasted duck protein compared to roasted ducks after SWS, indicating that VPS treatment promoted protein degradation [10]. It is worth noting that the apparent protein digestibility depends on the protein structures [11]. Hence, we speculated that VPS could change roasted duck breasts’ in vivo digestion properties.

Notably, most studies focused on the in vitro digestibility of meat protein, and research on the in vivo digestion properties still needs to be completed. Detailed research reports are indistinct, especially regarding the effect of the roasted duck after VPS on in vivo digestion properties and possible mechanisms. Therefore, the present study evaluated the effect of roasted duck after VPS on serum biochemical indicators (total protein, blood urea nitrogen, alkaline phosphatase, and blood glucose) and in vivo digestion properties (pepsin and trypsin activities and apparent protein digestibility) in rats. Additionally, the changes in the water distribution, textural properties, and protein structures of raw/roasted duck breasts were explored to explain how VPS affects in vivo digestion properties.

## 2. Materials and Methods

### 2.1. Materials and Chemicals

The thirty six-week-old male specific pathogen-free (SPF) grade Sprague–Dawley (SD) rats and the basal diet used in the experiment were purchased from Beijing Huafukang Bio-Technology Co., Ltd. (Beijing, China) (SYXK 2019-0022). The duck carcasses (two groups of six each, *n* = 12) were made from cherry valley ducks (41 days old, with an average weight of 1.3 ± 0.2 kg) and randomly selected from Hebei Dongfeng Breeding Co., Ltd. (Cangzhou, China). The duck carcasses were cleaned and packaged in nylon/polyethylene (Magic Seal, Dongguan, China) and chilled at −35 °C for 12 h before being transported to the laboratory via cold-chain logistics within 2 h.

The alkaline phosphatase assay kit, urea assay kit, glucose assay kit, and total protein assay kit were obtained from Shenzhen Rayto Life Science Co., Ltd. (Shenzhen, China). The pepsin activity assay kit and trypsin activity assay kit were obtained from the Nanjing Jiancheng Bioengineering Institute (Nanjing, China). All other reagents used were of analytical grade.

### 2.2. Processing of Salted Roasted Duck

The salted roasted ducks were prepared according to the method of Jiao et al. [5]. Static wet-salted duck carcasses and variable-pressure salted duck carcasses were roasted in a universal steam oven (Changsha Guli Food Machinery Co., Ltd., Changsha, China) for 50 min at 180 °C. The detailed processing conditions are shown in Table 1. The roasted duck breasts from the salted duck carcasses were minced using a knife mill. Then, the mixtures were freeze-dried, ground into powders, and stored at −80 °C.

### 2.3. Animal Treatment

The rats were housed in separate cages in a sterile room and fed water and a sesame-free diet at 23 ± 3 °C and 50 ± 10% relative humidity, with a 12 h light/dark cycle. The experiment protocol was approved by the animal ethics committee of Beijing Huafukang Bio-Technology Co., Ltd. (Beijing, China) (approval number: SYXK 2019-0022). The approval number from the animal ethics committee is HFK-AP-20221111.

### 2.4. Animal Experiment Design

Thirty SD rats (180 ± 20 g) were randomly divided into three groups (*n* = 10 rats per group) according to body weight: control check (CK), SWS-Roasted, and VPS-Roasted. The rats in the CK group were fed base diets. The rats in the SWS-Roasted group were fed diets containing 5% (m/m) static wet-salted roasted duck powder. The rats in the VPS-Roasted group were fed diets containing 5% (m/m) variable-pressure salted roasted duck powder. The diets were prepared according to the standard of Nutritional Composition of Combination Feed for Laboratory Animals (GB14924.3-2010) [12]. The composition of the diets is shown in Table S1.

### 2.5. Determination of Serum Biochemical Indicators in Rats

Total protein (TP), blood urea nitrogen (BUN), alkaline phosphatase (AP), and blood glucose (BG) contents were measured using a fully automated biochemical analyzer (Chemray 800, Rayto Life Science Co., Ltd., Shenzhen, China).

### 2.6. Analysis of In Vivo Digestion Properties in Rats

#### 2.6.1. Determination of Pepsin and Trypsin Activities

The pepsin and trypsin activities were determined according to the method described by Xu et al. [13]. Rat stomach tissue (1 mg) and small intestine (1 mg) were weighed, and 0.9% (m/v) saline (9 mL) was added. The mixture was homogenized under ice-bath conditions, and then the supernatant was centrifuged at 3000 r/min for 10 min. The pepsin and trypsin activities were determined in rats using pepsin and trypsin activity assay kits.

#### 2.6.2. Apparent Protein Digestibility Analysis

The protein content in the diet and feces of rats was determined with the method of Terrazas-Fierro et al. [14]. The apparent digestibility of protein was calculated as follows:

$$\mathrm{Apparent}\;\mathrm{protein}\;\mathrm{digestibility}\;(\%)=\frac{\mathrm{Protein}\;\mathrm{intake}\;-\;\mathrm{Fecal}\;\mathrm{protein}\;\mathrm{content}}{\mathrm{Protein}\;\mathrm{intake}}\times 100$$

### 2.7. Low-Field Nuclear Magnetic Resonance (LF-NMR) Analysis

The water distribution was measured following the method of Xu et al. [15] using an LF-NMR Analyzer (NMI20-040H-I, Niumag Electric Co., Ltd., Suzhou, China). Raw and roasted duck breast samples were cut into three strips (2 cm × 1 cm × 1 cm, weighing 5 ± 0.5 g) and placed in a test tube. *T*_2_ spectroscopy was conducted on the samples to determine transverse relaxation times using Carr–Purcell Meiboom–Gill (CPMG) sequences. The parameters settings were magnetic field strength: 0.5 T; proton resonance frequency: 23 MHz; SF: 23 MHz; P90: 9 μs; P180: 18 μs; TD: 59,990; TR: 3000 ms; NS: 16; and echo count: 2000. 

The H-proton density imaging spectra were obtained using the Nuclear Magnetic Resonance Imaging (NMI) system. The key parameters were repetition time: 2000 MS; performing: 4 repetition times; longitudinal relaxation time: 20 MS; and spin echo time: 20 MS.

### 2.8. Textural Properties and Warner–Bratzler Shear Force (WBSF) Testing

The textural properties were determined according to the method of Wang et al. [16] with minor modifications. The samples were cut into six cubes (1 cm × 1 cm × 1 cm) with a 1 ± 0.2 g mass. The measurement was conducted using a texture analyzer (TA-XT. Plus, Stable Micro Systems Co., Ltd., Godalming, UK) equipped with a P/50 column probe, a pretest speed of 2 mm/s, a test speed of 1 mm/s, and a test deformation of 50%. 

The WBSF was measured using the method of Jiao et al. [5]. The sample was cut into six strips (2 cm × 1 cm × 1 cm) according to the direction of the muscle fibers. The measurement was carried out using a texture analyzer (TA-XT. Plus, Stable Micro Systems, Godalming Surrey, UK) with a V-shaped blade slice–shear device, and an HDP BS probe at a pretest speed of 5 mm/s, a test speed of 2 mm/s, and a post-test speed of 10 mm/s. The maximum peak of the WBSF was recorded, and the WBSF is expressed in kilograms (Kg).

### 2.9. Analysis of Microstructure

The raw and roasted duck breasts were cut into cubes (3 cm × 3 cm × 2 mm) and then immersed in 25 mL/L glutaraldehyde for 24 h. The samples were washed three times with 0.1 mol/L phosphate buffer (pH 7.0). A series of ethanol concentrations (300 mL/L, 500 mL/L, 700 mL/L, 800 mL/L, 900 mL/L, 950 mL/L, and 1000 mL/L) were used for dehydration. The specimens were freeze-dried, sputter-coated, and then observed with a scanning electron microscope (SEM) (GeminiSEM 300, Carl Zeiss Co., Oberkochen, Germany).

### 2.10. Determination of Protein Structure

#### 2.10.1. Sodium Dodecyl Sulfate-Polyacrylamide Gel Electrophoresis (SDS-PAGE) Analysis

The SDS-PAGE analysis was performed according to Yin et al. [17] with slight modifications. Raw/roasted duck breast samples (2.0 g) of each treatment group were homogenized with 2% (*w*/*v*) SDS (10 mL) using a homogenizer (10,000 r/min, 3 × 30 s). The mixture was centrifuged at 4000× *g* (20 min), and the resulting supernatant was collected as the total protein extraction. The protein solution (2 mg/mL) was combined with a 5× loading buffer. Afterwards, the mixture was boiled at 100 °C for 10 min. Subsequently, the mixture (10 μL) was loaded in each lane of the precast gel (10% polyacrylamide). Electrophoresis was performed using a Mini-Protean Tetra System (Bio-Rad Laboratories, Hercules, CA, USA) under 80 V for 30 min, followed by 120 V for approximately 60 min. After separation, the gel was stained, decolored, and then photographed.

#### 2.10.2. Fourier Transform Infrared (FTIR) Spectroscopy Analysis

The protein secondary structure content was identified via a method delineated by Gangidi et al. [18] using a diamond-attenuated total reflectance (ATR) attachment (Cary 5000, Varian Co., Palo Alto, CA, USA). Raw/roasted duck breasts weighing 5.0 g were subjected to vacuum freeze-drying for 48 h and, subsequently, ground. Subsequently, the treated sample (0.5 g) was scanned. The scanning parameters were measuring range: 600~4000 cm^−1^; number of scans: 64; scanning rate: 0.63 cm/s; and resolution: 32 cm^−1^.

### 2.11. Statistical Analysis

All measurements were independently replicated at least three times with a completely randomized design. The results are presented as the mean ± standard deviation. The statistical analysis was conducted using ANOVA with SPSS 23.0 (SPSS Inc., Chicago, IL, USA), and the Duncan’s multiple range test was applied with a significance level set at *p* < 0.05. Data visualization and plotting were conducted using Origin software 2021 (Origin Lab Corporation, Northampton, MA, USA).

## 3. Results and Discussion

### 3.1. Analysis of Differences in Serum Biochemical Indicators in Rats

**Figure 1:**
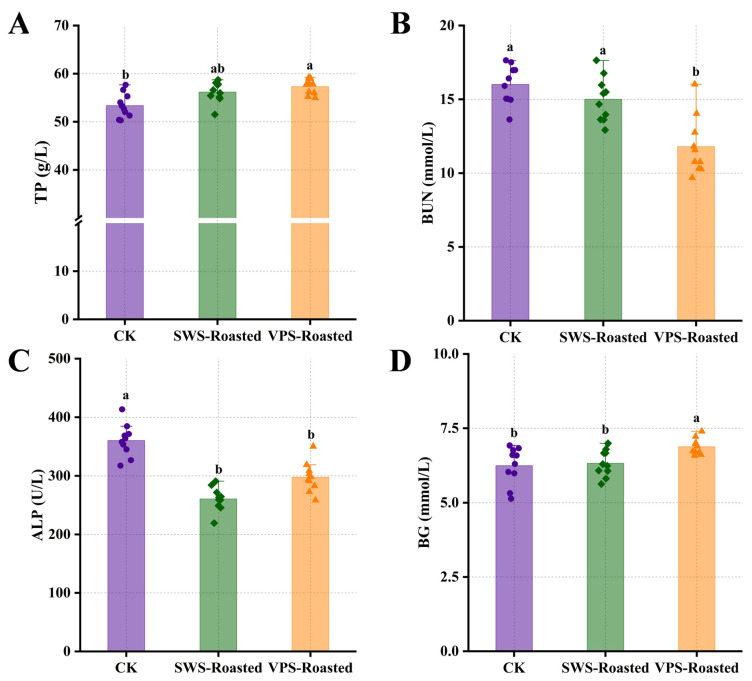
Effect of roasted duck treated with different saltings on total protein (TP) content (**A**), blood urea nitrogen (BUN) content (**B**), alkaline phosphatase (ALP) content (**C**), and blood glucose (BG) content (**D**) of rats. CK: base diet, SWS-Roasted: duck roasted after static wet salting, VPS-Roasted: duck roasted after variable pressure salting. Different letters indicate a significant difference (*p* < 0.05).

The total protein (TP) in serum serves various physiological functions, including maintaining blood osmotic pressure and pH and transporting metabolites [19]. Meanwhile, the TP is an essential source of protein for the body, reflecting its protein absorption [20]. As shown in Figure 1A, the TP levels in rats fed with duck breast roasted after VPS were significantly higher (*p* < 0.05) compared to the CK group. This observation demonstrates that duck breast roasted after VPS can promote protein absorption and precipitation in rats. The blood urea nitrogen (BUN) levels reflect the efficiency of the amino acid synthesis of proteins in the blood, with lower levels indicating enhanced protein anabolism and weakened catabolism in the animal body [21]. As presented in Figure 1B, the BUN content in rats fed with duck breast roasted after VPS was significantly lower than those in the CK and SWS-Roasted groups (*p* < 0.05), suggesting that duck breast roasted after VPS enhanced the protein anabolism in the rats in vivo, leading to a decrease in the synthesis of urea and a lower content of urea in the serum.

It is reported that a positive correlation between the alkaline phosphatase (ALP) contents and insulin resistance and high ALP activity may accelerate osteoblast metabolism and apoptosis [22]. The standard reference range for ALP content in rats is 12.04~610.97 U/L [23]. As observed in Figure 1C, the ALP contents of rats in all groups fell within the standard range. Notably, the ALP contents of duck breast roasted after VPS and SWS were significantly lower (*p* < 0.05) than that of the CK group, indicating that slow hepatic metabolism and bone metabolism may be present in rats. Moreover, in the standard range, an increase in blood glucose (BG) indicates that the body absorbs and metabolizes more nutrients [24]. In the fasting state, changes in BG are positively correlated with the body’s metabolism [25]. The BG contents of the rats in our experiment were measured in a fasting state. Thus, the primary source of BG was glucose production from the breakdown of hepatic glycogen from the liver [26]. As illustrated in Figure 1D, the highest BG content (6.93 mmol/L) was observed in the VPS-Roasted group, suggesting that the duck breast roasted after VPS could enhance the catabolism of liver glycogen in rats.

### 3.2. Differences in the Digestion Properties In Vivo

**Figure 2:**
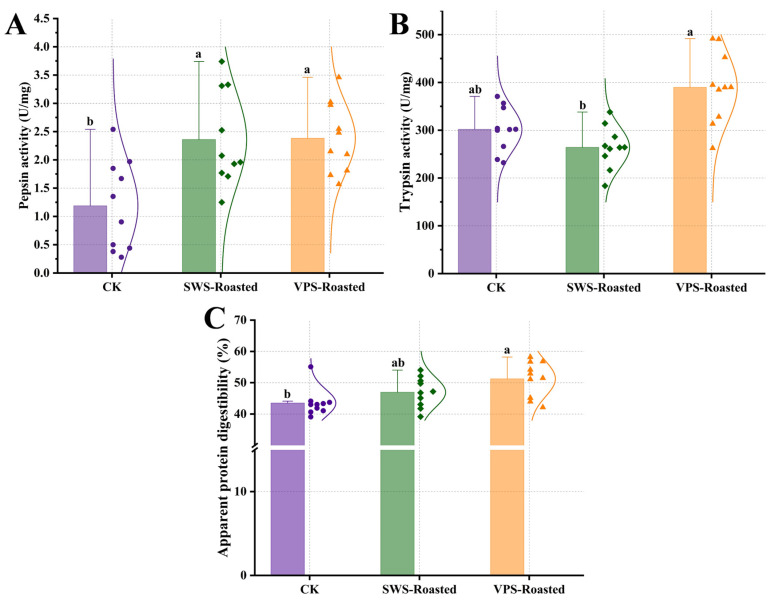
Effect of roasted duck treated with different saltings on the pepsin activity (**A**), trypsin activity (**B**), and apparent protein digestibility (**C**) in rats. CK: base diet; SWS-Roasted: duck roasted after static wet salting; VPS-Roasted: duck roasted after variable pressure salting. Different letters indicate a significant difference (*p* < 0.05).

Pepsin and trypsin are the predominant protein-digesting enzymes in the human body, and their activity can reflect the digestive capacity [27]. As shown in Figure 2A,B, there was a significant increase (*p* < 0.05) in pepsin activity in the rats fed duck breasts roasted after salting at variable pressure and static wet compared to the CK group. Additionally, the trypsin activity in rats fed duck breasts roasted after VPS was significantly higher than in the SWS-Roasted and CK groups (*p* < 0.05). These results indicate that duck breasts roasted after VPS increased the pepsin and trypsin activities in rats. Previous studies pointed out that the substrate concentration in the digestive tract determines the enzyme type and activity and that the substrate induces the secretion of relevant enzymes [28]. The highest feed intake in the rats (Table S2) fed duck breasts roasted after VPS increased the substrate for pepsin and trypsin actions and stimulated the gastric mucosa, thereby inducing the secretion of pepsin and trypsin.

The apparent protein digestibility is a critical indicator of the extent to which the products (amino acids and peptides) of food being digested are absorbed, and it depends on the protein structures [29]. Compared with the CK group, there was a significant increase (*p* < 0.05) in the apparent protein digestibility in the rats fed duck breasts roasted after VPS (Figure 2C). On the one hand, the duck protein and the soybean meal protein in the CK group contained different types, amounts, and spatial structures of amino acids, leading to differences in their protein properties and digestibility [30]. On the other hand, the VPS enabled raw meat to switch alternately between inhalation and extrusion of the curing solution, resulting in the increased dissociation of meat proteins and smaller molecular weights, facilitating better chewing and digestion [31].

### 3.3. Changes in Water Distribution

**Figure 3:**
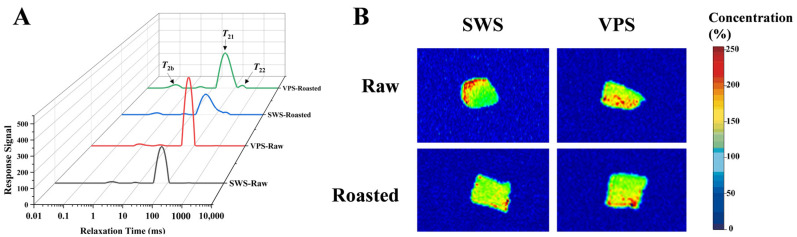
Effect of different salting treatments on the *T*_2_ transverse relaxation peak response signals (**A**) and H-proton imaging spectra (**B**) of the samples. SWS: static wet salting; VPS: variable pressure salting.

Low-field nuclear magnetic resonance (LF-NMR) reflects the migration pattern of water in different states in meat products by transverse relaxation times (*T*_2_) [32]. As observed in Figure 3A, from left to right, *T*_2b_ (0.1~10 ms), *T*_21_ (10~100 ms), and *T*_22_ (100~1000 ms) of relaxation represent bound water, immobilized water, and free water, respectively [33]. In addition, *P*_2b_, *P*_21_, and *P*_22_ represent the relative proportion of *T*_2b_, *T*_21_, and *T*_22_ relaxation areas, respectively (Table 2). By observing the change in the relative proportion, the relative water content in each state of the samples can be intuitively shown, thereby reflecting the change in water-holding capacity.

Immobilized water was the primary form of water in the samples and is positively correlated with water retention [34]. According to Table 2, the *P*_21_ was higher in the VPS-treated group than in the SWS-treated group (*p* < 0.05), suggesting that variable pressure salting improved the water-holding capacity of the duck breasts. The result was related to the salting effect of the VPS, which was efficient, with the higher ionic strength promoting the release of salt-soluble proteins and the formation of tighter three-dimensional protein network structures induced by heat treatment [35]. Moreover, compared with the raw breasts, the percentage of the free water relaxation area (*P*_22_) in the roasted samples increased significantly (*p* < 0.05). In contrast, the percentage of bound water relaxation area (*P*_2b_) and immobilized water relaxation area (*P*_21_) decreased significantly (*p* < 0.05). This phenomenon might be attributed to the weakening water–protein interaction caused by high heating and prolonged heating accelerating the movement of internal moisture out of the muscle tissue [36]. 

The distribution of H protons in the meat can be intuitively represented by the H-proton density imaging spectra [37]. The H-proton density is positively correlated with the moisture content of the samples, and the red areas in the image indicate the presence of proton [38]. As depicted in Figure 3B, the samples from the VPS treatments exhibited more prominent red images, indicating that more water was retained between the muscle fibers, and the water migration in the breasts was reduced [39]. It is noteworthy that in the SWS-Raw group, the H protons were mainly distributed at the border of the duck breast, whereas H protons spread to the middle of the muscle after VPS. This result further confirmed the homogeneity and effectiveness of variable pressure salting.

### 3.4. Changes in Textural Properties

**Figure 4:**
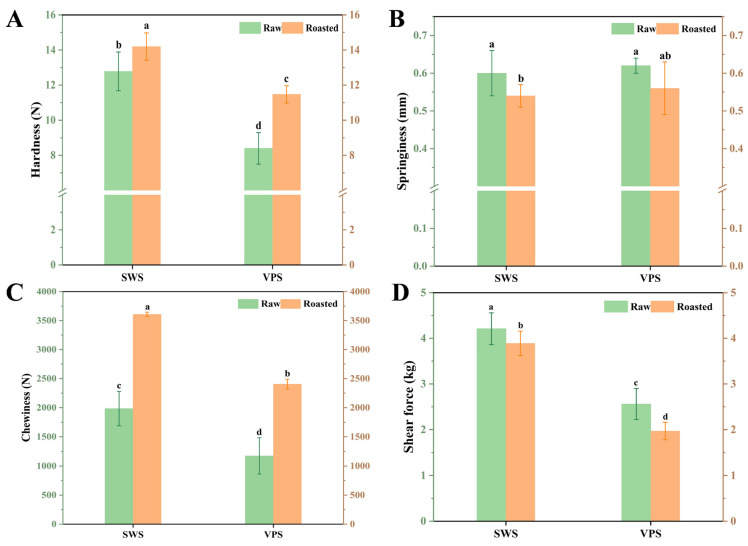
Effect of different salting treatments on the hardness (**A**), springiness (**B**), chewiness (**C**), and shear force (**D**) of the samples. SWS: static wet salting; VPS: variable pressure salting. Different letters indicate a significant difference (*p* < 0.05).

As shown in Figure 4, the textural properties of duck breast were significantly affected (*p* < 0.05) by roasting, VPS treatment, and their interaction. Compared to raw duck breast, the samples after roasting exhibited higher hardness values due to the contraction and dehydration caused by high-temperature heating. The hardness values of the VPS treatments were significantly lower than those of the SWS treatments (*p* < 0.05) (Figure 4A), indicating that VPS could improve the tenderness of the duck. Additionally, as presented in Figure 4B,C, the springiness values of the roasted duck breasts in the VPS treatments were higher than those in the SWS treatments, whereas the chewiness value was lower than that in the SWS treatment. These results might indicate that the springiness of the roasted duck due to VPS and the recovery after removing the external force were better than those of SWS. 

According to Figure 4D, the shear force in the VPS samples was significantly lower than in the SWS treatments (*p* < 0.05), which corresponded with Jiao’s findings [10]. Gao et al. [40] found that variable pressure tumbling was more effective than atmospheric pressure tumbling at reducing shear forces, possibly due to the changes in myofibrillar skeletal protein structure exacerbated by variable pressure. Moreover, Yang et al. [41] reported that high pressure regulates the degree of protein hydration with water to improve the tenderness of meat gel products. Consequently, it is plausible that VPS may improve the textural properties of duck breasts by altering the skeletal protein structures and increasing the content of immobilized water.

### 3.5. Analysis of Microstructure

**Figure 5:**
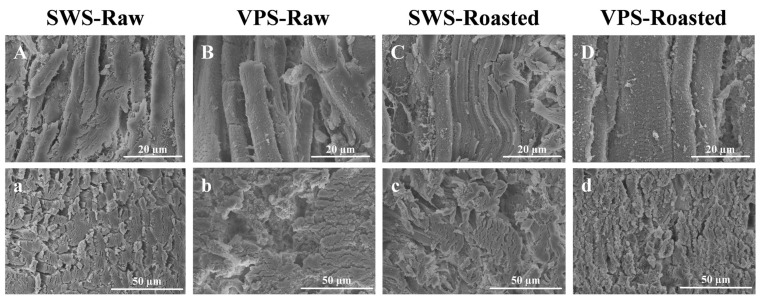
Light microscopic images of samples under different salting treatments. (**A**–**D**) Longitudinal sections of myofibrils of the SWS-Raw, VPS-Raw, SWS-Roasted, and VPS-Roasted treatments, respectively. (**a**–**d**) Transverse sections of myofibrils of the SWS-Raw, VPS-Raw, SWS-Roasted, and VPS-Roasted treatments, respectively.

The microstructure of the longitudinal and transverse sections of the samples treated with SWS and VPS is illustrated in Figure 5. The endomysium, which is elastic and maintains the integrity of the muscle, can rupture, indicating the loosening of muscle fiber bundles and an improvement in muscle tenderness [42]. As depicted, the endomysium of the duck breast muscle was destroyed after VPS, the muscle fiber gaps became extensive, and the tenderness improved. This result might be ascribed to the variable pressure expanding the internal space of myofibrils to retain more water [43]. In addition, compared to the raw duck treatments, the muscle fibers of the roasted duck treatments were significantly expanded by heat, resulting in a decreased distance between the muscle bundles, as well as increased hardness, as supported by the textural properties results (Figure 4). Notably, the diameter of the muscle fiber treated with VPS was more extensive than that of the SWS treatments, indicating that VPS could maintain higher levels of water retention and texture quality of the muscles. Therefore, VPS is an efficient salting method for improving the microstructure of the breast and may enhance its digestibility. 

### 3.6. Changes in Protein Structure

**Figure 6:**
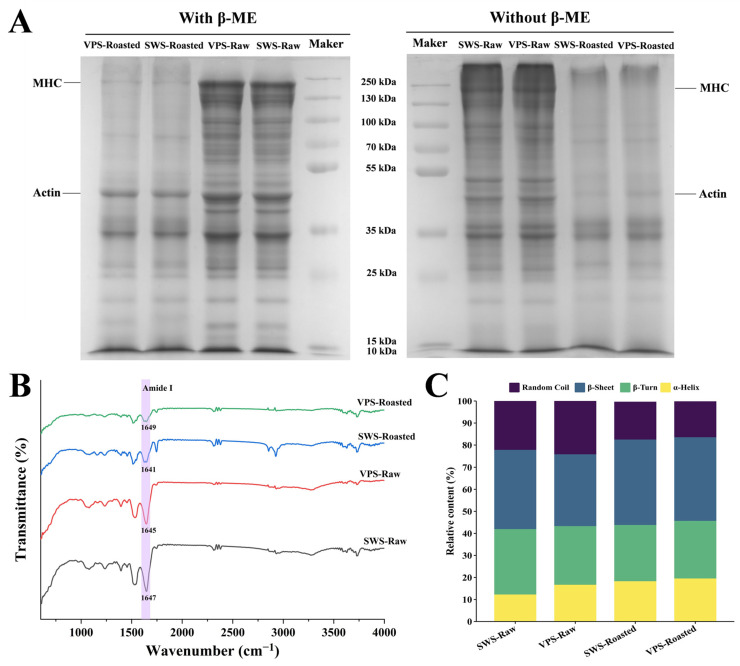
SDS-PAGE profiles (**A**) of protein in samples under different salting treatments. Proteins were prepared without and with β-mercaptoethanol (β-ME) addition. Changes in the infrared spectra (**B**) and relative protein secondary structure contents (**C**) under different salting treatments.

The reduced (with β-ME) and nonreduced (without β-ME) SDS-PAGE results of the total protein from duck breasts treated with VPS and SWS are depicted in Figure 6A. It is evident that the intensity of the protein bands for the roasted samples was weaker than those of the raw samples, indicating that the high temperature generated by roasting promoted the degradation of the duck breast protein [5]. Compared with the SWS-Raw treatment, the VPS-Raw treatment lanes’ bands deepened at 10~55 kDa. This result might be attributed to releasing some endogenous proteases in the muscle under the VPS treatment, facilitating protein solubilization and degradation. Cai et al. [44] also suggested that protein degradation mainly involves deepening at lower molecular weights and weakening bands at higher molecular weights. Furthermore, the intensity of the protein bands of the VPS-Roasted group was weaker than for the SWS-Roasted group. This phenomenon suggests that the protein degradation of duck breasts treated by VPS was more apparent after roasting, which makes it more suitable for digestion and absorption by the human body. 

As illustrated in Figure 6B, the peak shapes of the FTIR spectra curves were significantly altered after applying different salting methods. The amide I band (1600~1700 cm^−1^) is the main spectral band reflecting the secondary structure of proteins [45]. Higher contents of α-helixes and β-sheets reflect a more ordered structure, whereas more significant proportions of β-turns and random coils represent a more disordered structure [46]. As shown in Figure 6C, the total contents of α-helixes and β-sheets in the VPS-Raw group (49.27%) were higher than in the SWS-Raw group (48.15%), indicating that VPS could maintain the ordered structure of proteins. Moreover, Beattie et al. [47] found that the secondary structure of poorly tenderized muscle contained significantly more β-sheets than that of better-tenderized muscle. Interestingly, after roasting, the content of α-helix in the VPS-Roasted group (19.53%) was higher than in the SWS-Roasted group (18.27%), but the β-sheet content decreased (37.87%). This result implies that the duck breasts roasted after the VPS treatment had better tenderness than those treated with SWS, consistent with the textural properties (Figure 4). 

### 3.7. Correlation Analysis

**Figure 7:**
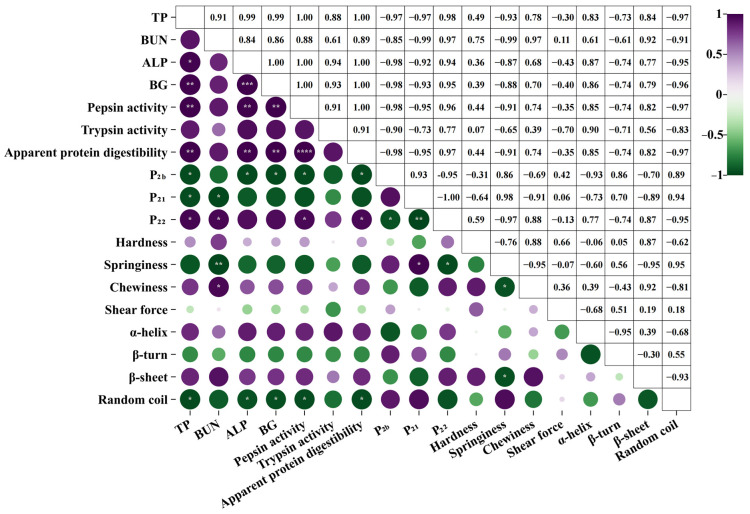
Heatmap of the correlation coefficients. The color of the scale bar denotes the nature of the correlation, with violet being a positive correlation and green being a negative. * *p* < 0.05, ** *p* < 0.01, *** *p* < 0.001, **** *p* < 0.0001.

The correlations between serum biochemical indicators, in vivo digestion properties, water distribution, textural quality, and protein structure are presented in Figure 7. The peak area of free water (*P*_22_), chewiness, and protein-ordered structure (α-helix and β-sheet) were found to have a positive correlation with serum biochemical indicators and in vivo digestion properties in the rats, indicating that roasted duck breasts with a higher water content, chewiness, and tenderness were more favorable for digestion and absorption in the body. Interestingly, there was no significant correlation (*p* > 0.05) between hardness, shear force, and in vivo digestion properties, which may be related to the fact that roasted duck breasts were mixed into the feed by stirring as powders. Furthermore, the bound water and immobilized water peak areas (*P*_2b_ and *P*_21_) of the roasted duck breasts showed a negative correlation with serum biochemical indicators and in vivo digestion properties of rats (*p* < 0.05). This result could be due to the transformation of bound and immobile water between muscle fibers to free water while preparing roasted duck breast into powders. In summary, the findings above demonstrate that the ordered structure of the protein, water content, and chewiness of the duck breasts roasted after VPS were superior to those roasted after SWS, which is more conducive for digestion in the body.

## 4. Conclusions

Our study demonstrates that VPS improves the texture quality of duck breasts by altering the protein structure and water distribution and enhancing the protein anabolism and in vivo digestion properties of rats that ingested VPS-treated roasted duck breasts. In detail, compared to SWS, the VPS-treated roasted duck breast promoted growth and serum biochemical metabolism and significantly increased the apparent protein digestibility, as well as pepsin and trypsin activities, in the rats. Furthermore, the VPS treatment improved the textural properties and microstructure of the duck breasts by regulating protein hydration with water, increasing the ordered protein structures and the immobilized water content to enhance the protein anabolism capacity and in vivo digestion properties in rats. Nevertheless, consuming VPS-treated roasted duck breasts should be moderated to control salt intake. In conclusion, the results initially confirm the feasibility of VPS-treated roasted duck breasts to improve the efficacy of digestion and absorption in rats. In the future, the effect of VPS-treated roasted duck breasts on rat intestinal health should be further investigated.

## Figures and Tables

**Table 1:** Processing parameters for salted roasted duck.

Parameters	Static Wet Salting	Variable Pressure Salting
Temperature/°C	4	4
Vacuum pressure/KPa	Atmospheric pressure	−70~−80
Mass fraction of salt solution/%	8.9	8.9
Pulse ratio	-	1.5 (54 min vacuum pressure/36 min atmospheric pressure)
Salting time/h	12	12
Roasting time/min	50	50

“-”: Indicates that the treatment was not carried out; atmospheric pressure: 101 KPa.

**Table 2:** Effect of different salting treatments on the *T*_2_ transverse relaxation peak area percentages (*P*_2_) of the samples.

Index/%	SWS-Raw	VPS-Raw	SWS-Roasted	VPS-Roasted
*P*_2b_	4.86 ± 0.95 ^a^	4.43 ± 0.25 ^a^	3.49 ± 0.20 ^b^	3.43 ± 0.37 ^b^
*P*_21_	94.91 ± 1.04 ^b^	95.30 ± 0.64 ^a^	89.26 ± 1.87 ^c^	90.75 ± 0.61 ^bc^
*P*_22_	0.23 ± 0.05 ^b^	0.27 ± 0.03 ^b^	7.25 ± 1.67 ^a^	5.82 ± 0.97 ^a^

SWS-Raw: duck after static wet salting; VPS-Raw: duck after variable pressure salting; SWS-Roasted: duck roasted after static wet salting; and VPS-Roasted: duck roasted after variable pressure salting. Different letters within the same row indicate statistically significant differences at a level of *p* < 0.05.

## Data Availability

The raw data supporting the conclusions of this article will be made available by the authors on request. The data are not publicly available due to privacy restrictions.

## Supplementary Materials

The following supporting information can be downloaded at: https://www.mdpi.com/article/10.3390/foods13030402/s1, Table S1. Diet composition and nutrient levels. Table S2. Effect of roasted duck treated with different salting on daily feed intake of rats (g/d).
