# Supplementary material for: Improving the Edible and Nutritional Quality of Roasted Duck Breasts through Variable Pressure Salting: Implications for Protein Anabolism and Digestion in Rats
Source: Foods. 2024 Jan 26;13(3):402. doi: 10.3390/foods13030402 (PMC10855416; doi:10.3390/foods13030402)
Supplement: Supplementary file 1 [file foods-13-00402-s001.zip › Supporting information.docx]

**Supporting Information**

**Table S1.** Diet composition and nutrient levels.

| **Composition (%, m/m)** | **CK** | **SWS-Roasted** | **VPS-Roasted** |
| --- | --- | --- | --- |
| Roasted duck powder | 0.00 | 5.00 | 5.00 |
| Yellow powder | 19.20 | 20.00 | 20.00 |
| Bran | 12.00 | 12.00 | 12.00 |
| Corn | 40.00 | 41.00 | 41.00 |
| Fish powder | 2.00 | 0.00 | 0.00 |
| Soybean meal | 20.80 | 16.00 | 16.00 |
| Soybean oil | 1.20 | 1.20 | 1.20 |
| Stone Powder | 1.52 | 1.52 | 1.52 |
| Calcium hydrogen phosphate | 2.00 | 2.00 | 2.00 |
| Salt | 0.40 | 0.40 | 0.40 |
| Mineral | 0.20 | 0.20 | 0.20 |
| Methionine | 0.20 | 0.20 | 0.20 |
| Choline | 0.40 | 0.40 | 0.40 |
| Vitamin | 0.08 | 0.08 | 0.08 |
| Total energy (Kcal/Kg) | 3868.00 | 3868.00 | 3868.00 |
| Protein content (%) | 21.70 | 21.70 | 21.70 |

CK: base diet, SWS-Roasted: duck roasted after static wet salting, VPS-Roasted: duck roasted after variable pressure salting.

**Table S2.** Effect of roasted duck treated with different salting on daily feed intake of rats (g/d).

| **Feeding times** | **CK** | **SWS-Roasted** | **VPS-Roasted** |
| --- | --- | --- | --- |
| One-week | 30.08 ± 2.52 ^c^ | 32.34 ± 1.33 ^b^ | 34.48 ± 1.56 ^a^ |
| Two-week | 30.79 ± 2.62 ^a^ | 31.96 ± 2.02 ^a^ | 33.13 ± 2.36 ^a^ |
| Three-week | 30.65 ± 3.23 ^a^ | 32.35 ± 2.38 ^a^ | 33.53 ± 2.92 ^a^ |
| Four-week | 30.23 ± 2.90 ^a^ | 31.47 ± 2.73 ^a^ | 32.83 ± 2.55 ^a^ |
| Average daily feed intake | 30.44 ± 2.62 ^c^ | 32.02 ± 2.08 ^b^ | 33.49 ± 2.19 ^a^ |

CK: base diet, SWS-Roasted: duck roasted after static wet salting, VPS-Roasted: duck roasted after variable pressure salting. Different letters (a ~ c) in the same row indicate a significant difference (*p* < 0.05).
