# Supplementary material for: Improving the Edible and Nutritional Quality of Roasted Duck Breasts through Variable Pressure Salting: Implications for Protein Anabolism and Digestion in Rats
Source: Foods. 2024 Jan 26;13(3):402. doi: 10.3390/foods13030402 (PMC10855416; doi:10.3390/foods13030402)
Supplement: Supplementary file 1 [file foods-13-00402-s001.zip › Supporting information.pdf]

# Supporting Information

**Table S1.** Diet composition and nutrient levels.

| Composition (%, m/m) | CK | SWS-Roasted | VPS-Roasted |
|---|---|---|---|
| Roasted duck powder | 0.00 | 5.00 | 5.00 |
| Yellow powder | 19.20 | 20.00 | 20.00 |
| Bran | 12.00 | 12.00 | 12.00 |
| Corn | 40.00 | 41.00 | 41.00 |
| Fish powder | 2.00 | 0.00 | 0.00 |
| Soybean meal | 20.80 | 16.00 | 16.00 |
| Soybean oil | 1.20 | 1.20 | 1.20 |
| Stone Powder | 1.52 | 1.52 | 1.52 |
| Calcium hydrogen phosphate | 2.00 | 2.00 | 2.00 |
| Salt | 0.40 | 0.40 | 0.40 |
| Mineral | 0.20 | 0.20 | 0.20 |
| Methionine | 0.20 | 0.20 | 0.20 |
| Choline | 0.40 | 0.40 | 0.40 |
| Vitamin | 0.08 | 0.08 | 0.08 |
| Total energy (Kcal/Kg) | 3868.00 | 3868.00 | 3868.00 |
| Protein content (%) | 21.70 | 21.70 | 21.70 |

CK: base diet, SWS-Roasted: duck roasted after static wet salting, VPS-Roasted: duck roasted after variable pressure salting.

**Table S2.** Effect of roasted duck treated with different salting on daily feed intake of rats (g/d).

| Feeding times | CK | SWS-Roasted | VPS-Roasted |
|---|---|---|---|
| One-week | $30.08 \pm 2.52^{c}$ | $32.34 \pm 1.33^{b}$ | $34.48 \pm 1.56^{a}$ |
| Two-week | $30.79 \pm 2.62^{a}$ | $31.96 \pm 2.02^{a}$ | $33.13 \pm 2.36^{a}$ |
| Three-week | $30.65 \pm 3.23^{a}$ | $32.35 \pm 2.38^{a}$ | $33.53 \pm 2.92^{a}$ |
| Four-week | $30.23 \pm 2.90^{a}$ | $31.47 \pm 2.73^{a}$ | $32.83 \pm 2.55^{a}$ |
| Average daily feed intake | $30.44 \pm 2.62^{c}$ | $32.02 \pm 2.08^{b}$ | $33.49 \pm 2.19^{a}$ |

CK: base diet, SWS-Roasted: duck roasted after static wet salting, VPS-Roasted: duck roasted after variable pressure salting. Different letters (a ~ c) in the same row indicate a significant difference ($p < 0.05$).
