# Supplementary figures and images for: Improving the Edible and Nutritional Quality of Roasted Duck Breasts through Variable Pressure Salting: Implications for Protein Anabolism and Digestion in Rats
Source: Foods. 2024 Jan 26;13(3):402. doi: 10.3390/foods13030402 (PMC10855416; doi:10.3390/foods13030402)

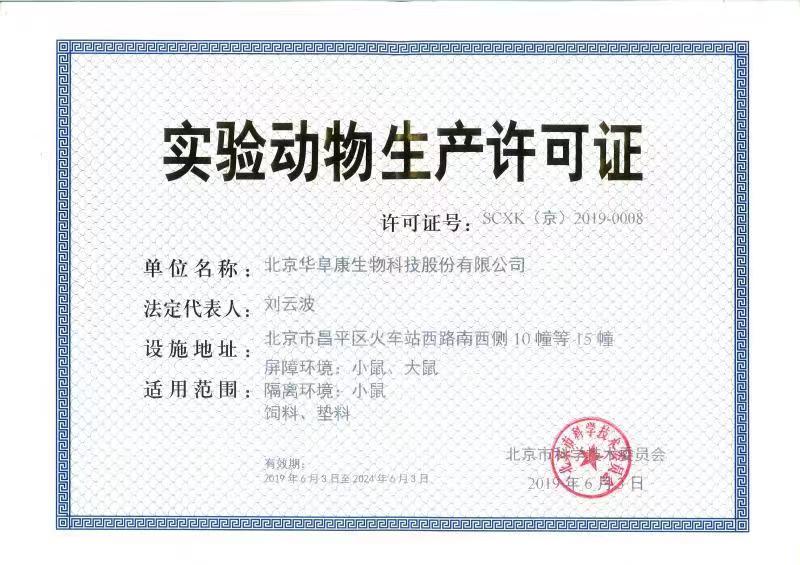

Supplement: Supplementary file 1 [file foods-13-00402-s001.zip › Laboratory animal production licence.jpg]

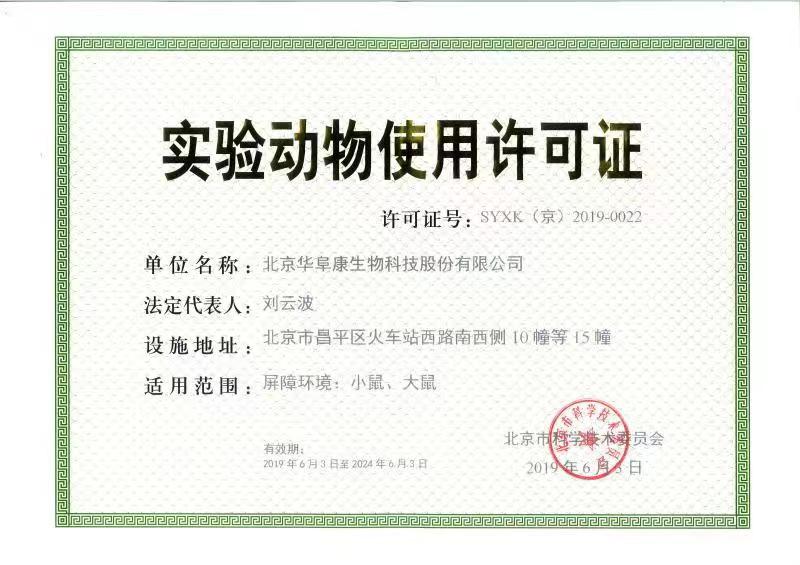

Supplement: Supplementary file 1 [file foods-13-00402-s001.zip › Licence for the use of laboratory animals.jpg]
